# Supplementary material for: Heparin-based hydrogel scaffolding alters the transcriptomic profile and increases the chemoresistance of MDA-MB-231 triple-negative breast cancer cells
Source: Biomater Sci. 2020 Feb 13;8(10):2786–96. doi: 10.1039/c9bm01481k (PMC7497406; doi:10.1039/c9bm01481k)
Supplement: Supplementary file 2 [file BM-008-C9BM01481K-s002.zip › Supplementary File 4/HP-B hydrogel + EGF (s)vControl/Pathways/my_analysis.Gsea.1545201170987/gsea_report_for_HYDEGF_1545201170987.html]

Report for HYDEGF 1545201170987 [GSEA]

| GS  follow link to MSigDB | GS DETAILS | SIZE | ES | NES | NOM p-val | FDR q-val | FWER p-val | RANK AT MAX | LEADING EDGE || 1 | HALLMARK\_MYC\_TARGETS\_V2 | Details ... | 56 | 0.50 | 1.87 | 0.000 | 0.000 | 0.001 | 4981 | tags=45%, list=26%, signal=60% |
| 2 | HALLMARK\_G2M\_CHECKPOINT | Details ... | 190 | 0.35 | 1.58 | 0.000 | 0.025 | 0.151 | 4319 | tags=33%, list=22%, signal=42% |
| 3 | HALLMARK\_MITOTIC\_SPINDLE | Details ... | 189 | 0.29 | 1.33 | 0.025 | 0.147 | 0.761 | 5633 | tags=41%, list=29%, signal=57% |
| 4 | HALLMARK\_E2F\_TARGETS | Details ... | 192 | 0.29 | 1.32 | 0.040 | 0.129 | 0.816 | 3814 | tags=24%, list=20%, signal=30% |
| 5 | HALLMARK\_TGF\_BETA\_SIGNALING | Details ... | 50 | 0.31 | 1.14 | 0.256 | 0.366 | 0.999 | 2027 | tags=24%, list=11%, signal=27% |
| 6 | HALLMARK\_MYOGENESIS | Details ... | 135 | 0.25 | 1.09 | 0.267 | 0.415 | 1.000 | 4286 | tags=32%, list=22%, signal=41% |
| 7 | HALLMARK\_SPERMATOGENESIS | Details ... | 76 | 0.26 | 1.00 | 0.417 | 0.568 | 1.000 | 3542 | tags=21%, list=18%, signal=26% |
| 8 | HALLMARK\_WNT\_BETA\_CATENIN\_SIGNALING | Details ... | 35 | 0.27 | 0.92 | 0.601 | 0.715 | 1.000 | 4592 | tags=37%, list=24%, signal=49% |
| 9 | HALLMARK\_KRAS\_SIGNALING\_DN | Details ... | 87 | 0.21 | 0.83 | 0.815 | 0.831 | 1.000 | 6140 | tags=38%, list=32%, signal=56% |
Table: Gene sets enriched in phenotype **HYDEGF (2 samples) **[plain text format]****
